# The authors’ response: Humanized Approach!

**DOI:** 10.5935/1518-0557.20140025

**Published:** 2014

**Authors:** Gabriella H. Vieira, Francisco A. C. Colucci

**Affiliations:** 1 Serviço de Reprodução Humana do Hospital Escola Alvaro Alvim - CIM-NF - Campos dos Goytacazes/RJ - Brazil

Dear Editor-in-Chief:

The article was made as a preliminary study of a bigger ongoing research, and it is important to take into consideration the aspects of both historical and social contexts of the studied patients: all of them resided in the Municipality of Campos dos Goytacazes, a city in the countryside of the State of Rio de Janeiro, at the time of the research, and found themselves in a moment in which the focus of their lives was to have a biological child. The study did not start from a deterministic perspective, psychologically speaking, so there is no fundamental characteristic in the patients, as we did not analyze the influence of pathologies or characteristics, but the final consequence of stress in each treatment.

The ISSL test (Lipp & Guevara 1994; Lipp 2000; SATEPSI 2014) used is referred and approved by the Federal Council of Psychology and it is apt for the private use of psychologists. It is recommended for researches related to stress in Brazil. The test was proposed by Marilda Lipp (Lipp & Guevara 1994, Lipp 2000), after 15 years of research in the Centro Psicológico de Controle de Estresse (Psychology Center of Stress Control) in Campinas, São Paulo. In which she proposed the fourfold model for stress, expanding from the threefold model that had been established so far, created by Hans Selye, in 1936. This way it is possible to evaluate the phases of: alertness, resistance, exhaustion, and near-exhaustion. The assessment tool ISSL consists of three scenarios that refer to the stress phases. The data collected due to applying the ISSL in the GI and GII groups were analyzed and compared in their stress level and verifying the phases of stress.

It is an instrument that has been validated since 1994 and has been used in several researches and clinical studies in the field of stress. It allows a diagnosis that evaluates if the person suffers from stress, in which phases it is, and if the stress manifests itself causing symptoms either physical or emotional. In the case study presented, the levels of stress were low in the exhaustion phase, which is considered the negative stress phase. And the objective was to verify the emotional consequences of each stimulus, and each treatment form. This was, it is understood that INVO offers patients a more personal approach to achieve pregnancy; generating the involvement the patients seek while on treatment (Carvalho *et al.*, 2006), resulting in a less cold and more humanized treatment.
